# Novel Nanotechnological Approaches for Targeting Dorsal Root Ganglion (DRG) in Mitigating Diabetic Neuropathic Pain (DNP)

**DOI:** 10.3389/fendo.2021.790747

**Published:** 2022-02-08

**Authors:** Ranjana Bhandari, Ashmita Sharma, Anurag Kuhad

**Affiliations:** University Institute of Pharmaceutical Sciences, Panjab University, Chandigarh, India

**Keywords:** diabetic neuropathic pain (DNP), nanotechnology, dorsal root ganglion (DRG), siRNA, extracellular vesicles, ligand-based targeting, nanoparticles

## Abstract

Diabetic neuropathy is the most entrenched complication of diabetes. Usually, it affects the distal foot and toes, which then gradually approaches the lower part of the legs. Diabetic foot ulcer (DFU) could be one of the worst complications of diabetes mellitus. Long-term diabetes leads to hyperglycemia, which is the utmost contributor to neuropathic pain. Hyperglycemia causing an upregulation of voltage-gated sodium channels in the dorsal root ganglion (DRG) was often observed in models of neuropathic pain. DRG opening frequency increases intracellular sodium ion levels, which further causes increased calcium channel opening and stimulates other pathways leading to diabetic peripheral neuropathy (DPN). Currently, pain due to diabetic neuropathy is managed *via* antidepressants, opioids, gamma-aminobutyric acid (GABA) analogs, and topical agents such as capsaicin. Despite the availability of various treatment strategies, the percentage of patients achieving adequate pain relief remains low. Many factors contribute to this condition, such as lack of specificity and adverse effects such as light-headedness, languidness, and multiple daily doses. Therefore, nanotechnology outperforms in every aspect, providing several benefits compared to traditional therapy such as site-specific and targeted drug delivery. Nanotechnology is the branch of science that deals with the development of nanoscale materials and products, even smaller than 100 nm. Carriers can improve their efficacy with reduced side effects by incorporating drugs into the novel delivery systems. Thus, the utilization of nanotechnological approaches such as nanoparticles, polymeric nanoparticles, inorganic nanoparticles, lipid nanoparticles, gene therapy (siRNA and miRNA), and extracellular vesicles can extensively contribute to relieving neuropathic pain.

**Graphical Abstract:**
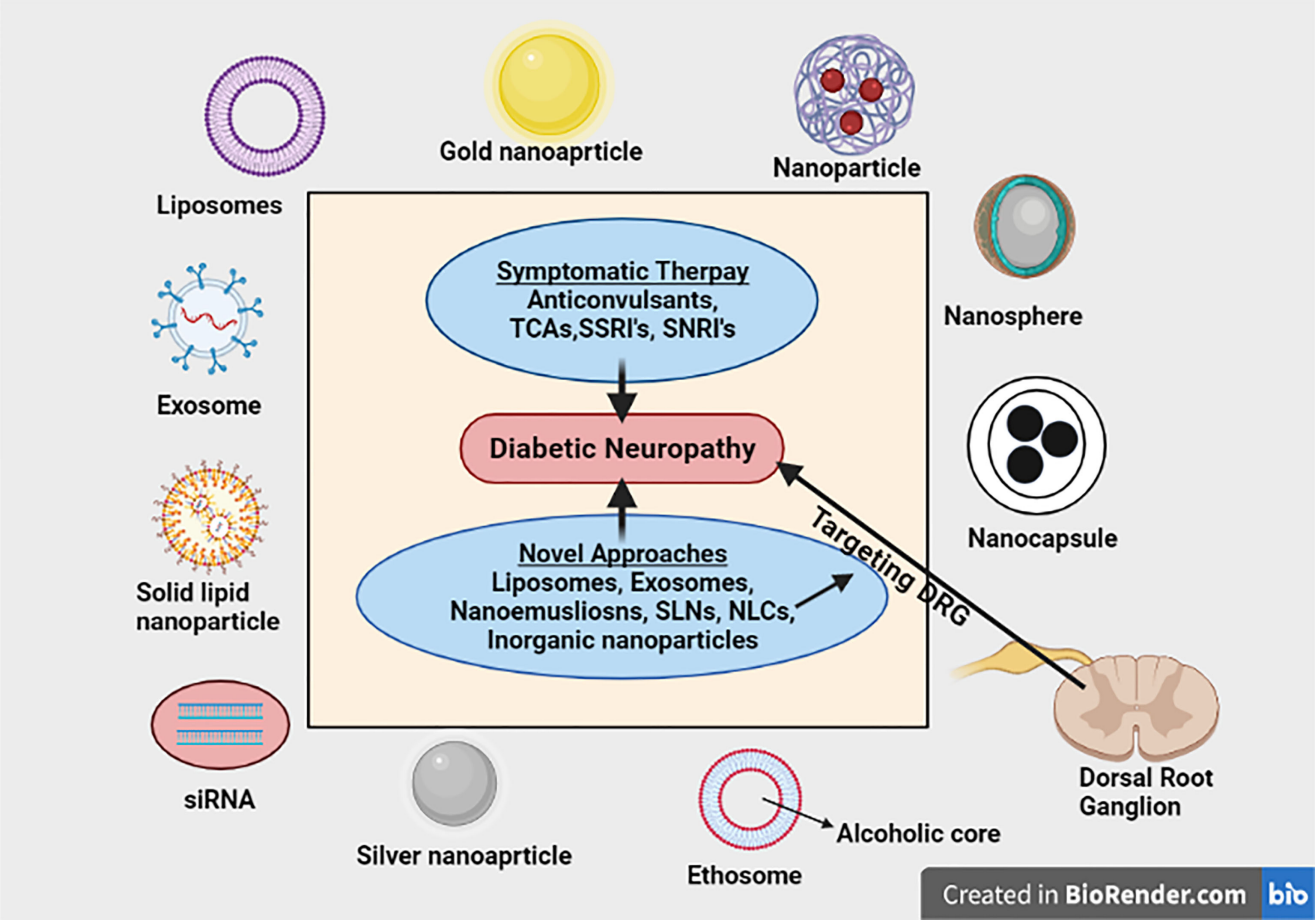
Nanotechnology based strategies has been extensively studied for their potential application in improving the delivery of drugs mitigating neuropathic pain to the targeted area with enhanced action. This review has comprehensively summarized and critically discussed the application of various novel nanotechnological approaches for mitigating diabetic neuropathic pain specifically targeting DRG.

## Highlights

- Diabetes mellitus is a common metabolic disorder characterized by diabetic neuropathy, influencing around 90% of patients.
- Symptoms of diabetic neuropathic pain become unpleasant and disturbing at night and usually involves burning sensation, acute cricking, plunging, and body aches, especially in the lower part.
- Studies suggest that the dorsal root ganglion (DRG) is an active participant in peripheral processes, including platelet activation factor (PAF) damage, inflammation, and the production of neuropathic pain.
- Nanotechnology plays a significant role in effectively delivering drugs (analgesics) to specific sites, thus mitigating chronic pain.

## 1 Introduction

Diabetes mellitus (DM) is a common metabolic disorder characterized by diabetic neuropathy, influencing around 90% of patients (1). Neuropathy develops gradually, usually after 25 years of disease (2). The pervasiveness of painful diabetic neuropathy (PDN) ranges from 10% to 20% in diabetics (3). In 2020, approximately 34.2 million people had diabetes, and out of that, around 26.9 million people, including adults, were undiagnosed (4). Distal symmetrical peripheral neuropathy (DSPN) is the most dominant form of diabetic neuropathy, affecting 20% of type-I diabetic patients worldwide. Normally, it affects the distal foot and toes, which gradually approaches the lower part of the legs (5). The clinical manifestations of DSPN include foot ulceration and serious neuropathic pain (5). Symptoms of diabetic neuropathic pain (DNP) become unpleasant and disturbing at night and usually involves burning sensation, acute cricking, plunging, and body aches, especially in the lower part. Sometimes, diabetic neuropathy leads to neuropathic cachexia, accompanied by depression and loss of weight (1). Many apparent mechanisms have been put forward to elucidate the pain related to diabetic neuropathy, including auto-oxidative stress, hyperglycemia, agitated polyol pathway, enhanced levels of advanced glycation end products (AGEs), and rise in protein kinase C (PKC) (mainly β-isoform). As compared to nerves, dorsal root ganglion (DRG) is more assailable to oxidative stress (6). Recent studies have shown that DRG neurons offer a plausible target and are linked with various problems of diabetic neuropathy (6). DRG possesses many applications, particularly for DNP (7). Nowadays, the stimulation of DRG has been considered a new neuromodulation paradigm. Various techniques are being employed or utilized for DRG stimulation, but implantable devices are gaining recognition to a greater extent (8). DRG neurons emerge from the dorsal root of spinal nerves, conveying sensory signals to the central nervous system (CNS) for a response to various receptors (9). Studies suggest that DRG is an active participant in peripheral processes, including platelet activation factor (PAF) damage, inflammation, and the production of neuropathic pain (9). Peripheral damage to the nerves in neuropathic pain leads to overexpression of the P2X3 receptor in the DRG (10). Apart from the P2X3 receptor, studies suggested that the P2X4 receptor also plays a key role in neuropathic pain. DRG consists of satellite glial cells (SGCs), which are mainly involved in the expression of the P2X4 receptor. Whenever there is nerve impairment, it is accompanied by the liberation of ATP, which further stimulates P2X4 receptors on SGCs, thereby generating neuropathic pain (11). Transient receptor potential vanilloid (TRPV) is also concerned with DNP, as it plays a vital role in nociceptive transference under clinical forms of pain (11, 12). The primary key in controlling DNP is symptomatic treatment (13). Various drugs have been recommended to minimize neuropathic pain either alone or in combination. The USA has given regulatory allowance to three drugs in treating DNP: pregabalin, duloxetine, and tapentadol (5). Although there are numerous therapeutic agents utilized in the treatment of DNP, half of the population is not able to achieve adequate pain relief. This failure is not due to the lack of efficacy of the drug but due to inadequate drug delivery at the site of action (14). Therefore, we need to incorporate innovative drug delivery systems to overcome the limitations offered by conventional ones. Nanotechnology plays a major role in effectively delivering drugs (analgesics) to specific sites, thus mitigating chronic pain. The main drawback offered by analgesics was their toxicity; thus, incorporating them into nanocarriers greatly enhanced their efficacy and reduced their toxicity. Some of the common analgesics, namely, baclofen, bupivacaine, and morphine, were formulated with liposomes, polyesters, poly (lactic-co-glycolic acid) (PLGA), nanoemulsions, etc., to improve their efficacy (11). It is reported that P2X3 receptor activation leads to allodynia in rat models of diabetes (15). DM rats, when treated with NONRATT021972 [long non-protein-coding RNAs (lncRNAs) siRNA], have shown that the expression of the DRG P2X3 receptor is significantly decreased as compared to type 2 diabetes mellitus (T2DM) rats in which no treatment is given. Unlike aqueous drugs, baclofen-loaded PLGA nanoparticles enhanced the retention duration of drug in the brain in order to mitigate neuropathic pain and turned out to be a suitable carrier for baclofen (16). Similarly, another emerging technology involves ribonucleic acid interference (RNAi) that mainly blocks gene assertion after transcription. Due to this inhibition, there is stimulation of RNA-induced silencing complex (RISC), which further hampers the protein synthesis. Potential benefits of bupivacaine were analyzed after its local delivery in people suffering from constant DRG compression (17). In the following review, novel approaches for targeting the DRG with the illustration of physiology of DRG and pathophysiology of DNP are discussed.

### 1.1 Epidemiology

One of the most recognized complications of DM is DNP. In various studies across India, PN prevalence ranges from about 10.5% to 32.2% in diabetic patients (18). Compared to the West, it has a higher prevalence of DM in India (4). Nowadays, practically in every country, diabetes impacts the population and increases medical load. Diabetes has become an epidemic globally; nearly 463 million adults in the age group of 20–79 years had diabetes in 2019, and this number is projected to grow to 700 million by 2045 (19). In Indian epidemiological studies from different areas, the average prevalence of PN in various community studies ranged from 5 to 2,400 per 10,000 population (20). Pain is one of the most pronounced symptoms of diabetic polyneuropathy. The incidence of diabetic peripheral neuropathy (DPN) was 46% in the African population in a survey conducted in 2020. Apart from this, the highest prevalence was reported in West Africa, accounting for about 49.4% (21). In autonomic neuropathy, the extent to which symptoms occur is relatively low (0%–10%), except impotence, whose chances of occurrence are about 5%–50% (22). As per reports from Europe and the USA in the year 2007, it has been revealed that the prevalence of DPN ranges from 6% to 51% with successive years of follow-up (13–14 years) (23). The pervasiveness of DPN in adults increased to 30% from 6% in type 1 diabetic patients as per the study conducted by Diabetes Control and Complications Trial/Epidemiology of Diabetes Interventions and Complications (DCCT/EDIC) (24). A survey from the Consensus Development Conference on Diabetic Foot Wound Care suggested that around 26% of youth with type 2 diabetes developed DPN, thereby concluding that type 2 diabetics are more prone to develop neuropathic pain (25). Foot ulceration is one of the common manifestations of diabetic neuropathy. In some patients (14%–24%), foot ulceration is so severe that, sooner or later, it requires amputation (26). Patients with a previous history of foot ulcers, foot malformation, poor sugar control, smoking, etc., are at higher risk of amputation (27). Older adults are more prone to diabetic neuropathy who have had chronic diabetes for a long time (28). Some studies demonstrated that diabetic neuropathy is less observed in the Asian population, although there was no evidence or finding supporting this particular statement (29). More recently, DPN’s prevalence has been reevaluated in young people with shorter durations of diabetes.

### 1.2 Physiology of Dorsal Root Ganglion

**Figure 1:**
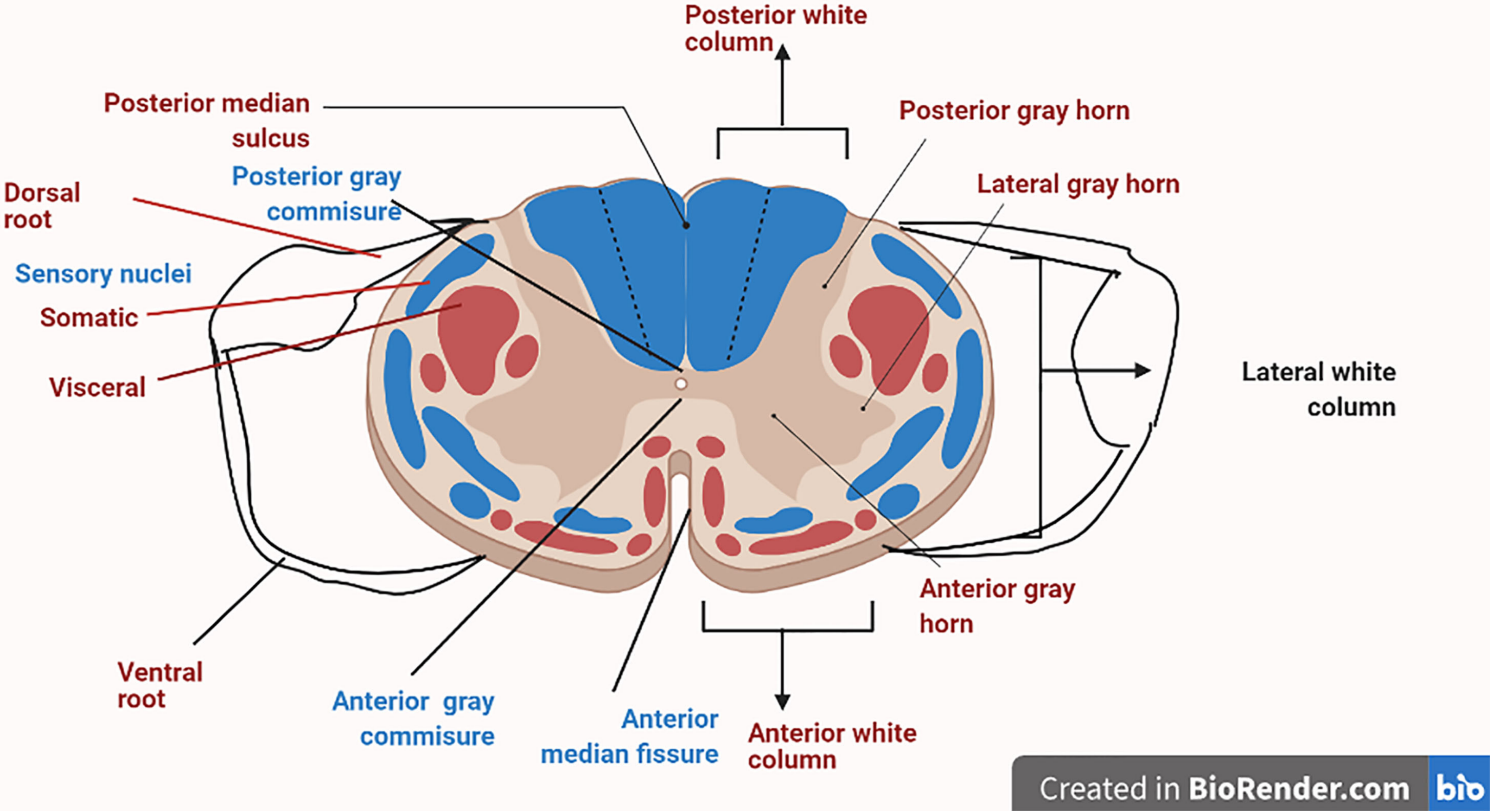
Sectional organization of the spinal cord showing the dorsal root ganglion.

DRG is one of the most condemning structure in sensory signaling and modulation, along with pain transmission (30). A very thin boundary of cerebrospinal fluid (CSF) surrounds the sural sheath in which DRG is located (31). DRG is a mere extension of the dorsal root that usually accommodates cell bodies of primary sensory neurons (PSNs). The diameters of cell bodies can be classified as large-light neurons (which are generally known as A-neurons, and these usually transmit non-noxious information) or small-dark neurons (traditionally known as C-neurons, which transmit painful signals) (32). The axon soon gets bifurcated into a T-like fashion into a peripheral branch, which is connected with somatic and visceral receptors, and finally enters into a central component that ends up into a cord (33). The DRG’s root sheath covers the dorsal root cord and traverses the subarachnoid space toward it. The proximal part usually consists of numerous tiny rootlets entering the dorsolateral cord in a defined manner (33). The DRG central projections typically end up in the corresponding segment. DRG is in close association with the sympathetic chain *via rami communicantes* nerves. Sometimes, these nerves can act as channels for discogenic afferents that can deliver spinal pain signals to the DRG (34) (**Figure 1**).

#### 1.2.1 Changes in Spinal Cord

The increased spinal neuronal activity due to neuropathy can be linked to the enhanced activity of the dorsal horns (35). In animal models having nerve injury, animal models have the stimulation of various protein kinases protein kinase A (PKA), p38, Src, extracellular signal-regulated kinase (ERK), calcium/calmodulin-dependent protein kinase (CaMK)II, and mitogen-activated protein kinase (MAPK). In addition, many CNS changes are associated with the production of inflammatory mediators. For example, the dorsal horn neurons possess increased expression of chemokines such as SDF-1α/CXCL12 and CXCL13 in rat models (36).

#### 1.2.2 Initial Pathological Changes in the Dorsal Root Ganglion (Primary Triggers for Neuropathic Pain)

Major changes in primary sensory neurons altered gene/protein expression. Due to the destruction of peripheral sensory fibers, hyperalgesia occurs due to upregulation in the face of Cav α2 δ-_1_ channel subunit, the Nav 1.3 sodium channel, and bradykinin (BK) B1 and capsaicin TRPV1 receptors (37). In addition, there is an immense increase in the expression of neurotrophic factors such as nerve growth factor (NGF) and neurotrophin-3 (NT-3). These neurotrophins are present in satellite glial cells (SGCs), which usually surround neuron cell bodies in DRG (38).

### 1.3 Pathophysiology of Diabetic Neuropathic Pain

The pathophysiology of DNP is quite complex, and there is no complete evidence to understand it entirely. Arteries endowing peripheral nerves undergo numerous changes and have been considered one reason behind aches and pains related to diabetic neuropathy. Recently, variations in sodium and calcium channel expression and central pain mechanisms have been linked to pain (39). Moreover, DNP is manifested by various risk factors, including old age, smoking, alcohol intake, and long-term diabetes (40). Due to a reduction in heat- and cold-specific C fibers and Aδ fibers, respectively, neuropathy leads to cold and heat allodynia (41). Mitochondria malfunctioning causes many problems in the body such as induction of neuropathic pain and changes in the peripheral nervous system (42). Various mitochondrial mechanisms, including calcium regulation (43), production of reactive oxygen species (ROS) (44), and apoptotic signaling pathways (45), are significantly involved in the development of neuropathic pain. Therefore, it is not only one single pathway that causes pain, but so many interconnected pathways operate together to start the cascade leading to neuropathic pain.

Changes in sodium channel expression appear to be triggered by hyperglycemia. In pain models of neuropathy, upregulated sodium channels (voltage-gated) were commonly seen in the DRG (46). Impairment of Na^(+)^-K^(+)^ pump occurs basically due to hyperglycemia, and it affects Na^(+)^ currents to a great extent (47). Along with their transmission, these channels impact action potential processing and can be regarded as tetrodotoxin sensitive (TTX-S) (48). Tetrodotoxin-sensitive Nav1.3 channels are usually upregulated in diabetic animal models (49) and Nav1.7 in the DRG (50) (51, 52). Na^+^ channels are repeatedly opened due to sensory neurons of DRGs, and their opening duration has also been seen to be prolonged to elevate the levels of intracellular sodium ions. Due to polarization of the neuron, there is increased opening of calcium channels that further leads to hyperpolarization (47). Rats in which nerves of the spinal area are injured show the oversensitivity of nociceptive responses to harmless mechanical stimulation due to overexpression of α2δ-1 subunit of the calcium channel (53). Due to this overexpression, more calcium enters the cell, leading to various signaling cascades (53). Also, the release of glutamate in the presynaptic zone leads to stimulation of N-methyl-D-aspartate (NMDA) receptors. This activation of the NMDA receptor will enhance the influx of calcium into the cell, thus rising calcium levels intracellularly (54). In response to the hyperpolarization of cells, mitochondria start releasing more calcium in the cytoplasm from its intercellular stores. As calcium concentration elevates inside the cell, it leads to activation of various signaling cascades mainly involving phosphorylation of PKC (55), causing an upregulation of TRPV (56), which directly causes variations in the sensory neurons, which result in a hyperresponsive state. There is the generation of nitrogen and oxygen-free radicals due to the upregulation of TRPV, leading to neuronal cytotoxicity (57).

**Figure 2:**
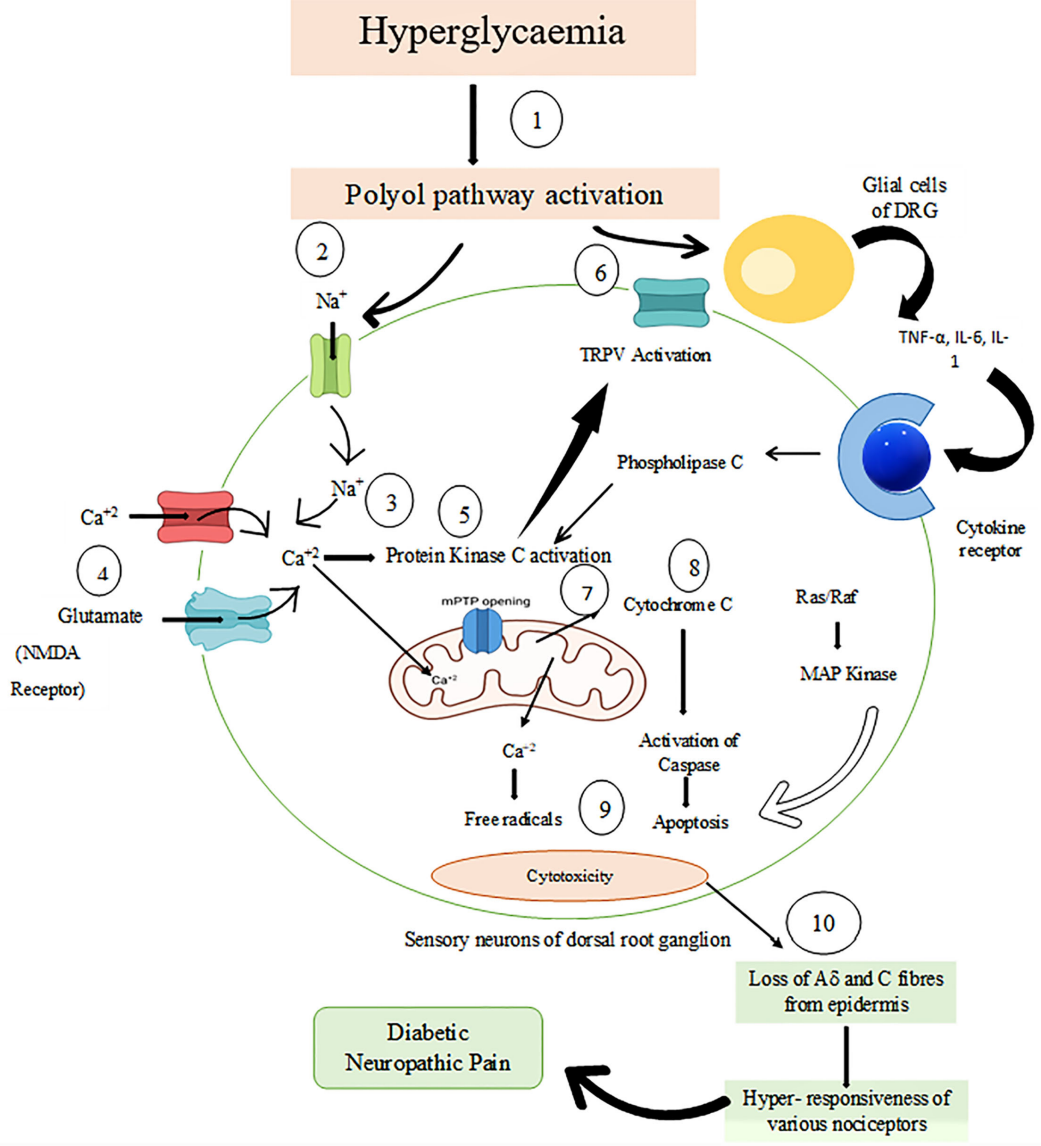
Pathophysiology of diabetic neuropathic pain. 1) Hyperglycemia stimulates the polyol pathway, which leads to the destruction of sodium currents. 2) Na^+^ channels were repeatedly opened due to sensory neurons of DRG, thus leading to increased sodium ions intracellularly. 3) As a result of polarization, there is the further opening of calcium channels. 4) In the presynaptic zone, glutamate causes activation of NMDA receptors and enhances the entry of calcium. Due to increased calcium levels, this triggers more calcium release from mitochondrial stores. 5) Activation of protein kinase C takes place due to increased calcium levels. 6) Transient receptor potential vanilloid (TRPV) phosphorylation and activation occur *via* protein kinase C due to which sensory neurons become hyperresponsive and also there is ROS and nitrogen radical generation, which causes cellular toxicity. 7) After the opening of mPTPs, there is the release of cytochrome C, 8) which initiates apoptotic avalanche with activation of caspases leading to sensory neuronal destruction 9) and finally leads to apoptosis. 10) Epidermis may lose some Aδ and C fibers, which causes hyperresponsiveness of various nociceptors. Inflammatory mediators such as IL-1, IL-6, and TNF-α are also involved in this process and play an essential role in developing neuropathic pain. DRG, Dorsal Root Ganglion; NMDA, N-methyl-D-aspartate; TRPV, Transient Receptor Potential Vanilloid; ROS, Reactive Oxygen Species; mPTP, mitochondrial permeability transition pores; IL-6 & IL-1, Interleukin 1 & 6; TNF-alpha, Tumour Necrosis Factor-alpha.

TRPV1 coresides with transient receptor potential ankyrin 1 (TRPA1) in particular neurons of DRG, and it is proven to have a role in the generation of the pain signals and in inflammation that may occur due to various irritants such as chemical agents, ROS, or nitrogen radicals (58). Increased permeability of mitochondrial permeability transition pores (mPTPs) due to hyperpolarization inside the neuronal body may cause the release of cytochrome C that further begins apoptotic cascades. During the apoptotic pathways, caspases get activated, which can cause the destruction of neuronal bodies and can cause cellular toxicity (59). Consequently, the number of cold-specific Aδ fibers and heat-specific C fibers starts reducing from the epidermis, known as loss of intraepidermal nerve fibers, and loss of nociceptors has also been observed that will result in the hyperresponsive state of the remaining nociceptors (60). Various inflammatory mediators involving tumor necrosis factor (TNF)-α, interleukin-1 (IL-1), and IL-6 are also seen to be involved in this signaling cascade. Cytokines, after binding to their receptors, lead to activation of PKC and MAPK that further corresponds to the development of neuropathic pain (61). These inflammatory cytokines usually enhance the expression of various ion channels involving sodium channels that causes neuronal excitotoxicity and significantly contributes to neuropathic pain pathogenesis (62). The pathogenesis of DNP interconnecting different pathways is represented in **Figure 2**.

#### 1.3.1 Oxidative and Nitrosative Stress

The main trigger for the generation of oxidative stress in DNP could be activating the polyol pathway. However, some other factors can also contribute to the initiation of oxidative stress such as glucose auto-oxidation, rise in AGE levels intracellularly, enhanced expression of AGE receptors, and at last hyperactivity hexosamine pathway. Some evidence highly supports the fact that there is generation of oxidative stress due to glucose metabolism itself. Apart from oxidative stress, another key player that plays a crucial role in the development of diabetic complications is reactive nitrogen species, mainly peroxynitrite. In the animal models of diabetes, it has been observed that there are insignificant tissue concentrations of carbonyl compounds. The catalase and glutathione levels are precisely the same in DNP patients compared with non-diabetic neuropathic ones. This oxidative stress plays a significant role in the generation of chronic pain mechanisms and DNP (5).

#### 1.3.2 Pro-Inflammatory Signaling in Diabetic Neuropathic Pain

The progression of diabetic neuropathy is associated with the acquirement of the pro-inflammatory process endured by nerve tissues. There is enhanced nerve conduction velocity (NCV) delay due to cytokine release hindrance and macrophage migration inhibition. The innate immune system is triggered by low-grade inflammation and plays a vital role in the pathogenesis of DNP. Inflammation is arbitrated by protein high-mobility group box 1 (HMGB1) released by immune cells. HMGB1 signaling was considered as one of the most poorly regulated pathways. This observation was made while comparing the differentially expressed genes between diabetic and non-diabetic patients. HMGB1 signaling is induced *via* Receptor for advanced glycation endproducts (RAGE) and Toll-like receptors (TLRs), both of which are involved in DPN. Therefore, these dysregulations of pathways linked with transcription implicate a lot in the pathophysiology of DPN (63).

#### 1.3.3 Pharmacogenetic Analysis of Diabetic Neuropathic Pain

A genome-wide association study (GWAS) was conducted to determine the genetic contributors involved in DNP. The study involved monitoring patients having neuropathic pain consuming at least one of the five drugs [duloxetine, gabapentin, pregabalin, capsaicin cream (or patch) and lidocaine patch] indicated. However, diabetic individuals with no history of consuming these drugs were taken as control. Individuals who had a prescription history of amitriptyline, carbamazepine, or nortriptyline were not included as controls because these drugs are often used for the treatment of other medical conditions, as well as neuropathic pain. After the successful analysis, it was observed that sex-specific narrow sense heritability was higher in males (30.0%) as compared to females (14.7%). This specific GWAS analysis provides ample evidence about the involvement of sex-specific Chr1p35.1 (ZSCAN 20-TLR 12P) and Chr8p23.1 (HMGB1P46) in DNP. Here, abbreviations has been explained of ZSCAN 20-TLR. Zinc finger and SCAN domain containing 20 (ZSCAN20), TLRs, HMGB1 (64).

Another study evaluated the impact of *CYP2D6* genotype on amitriptyline efficacy for the treatment of DPN. Randomly, 31 participants were selected and given low-dose amitriptyline, and after some time, their *CYP2D6* gene was sequenced. As a result, fewer side effects were observed in patients possessing ultrarapid metabolizer phenotypes. Therefore, this study can guide drug therapy for DNP shortly (65). There are numerous drugs in the market for the treatment of neuropathic pain. Furthermore, we describe competitive market landscape, market potential, and limitations of current therapy.

#### 1.3.4 Protein–Protein Interaction

Having a deep insight of molecular mechanisms associated with a particular disease is the foremost goal of modern medical research. In order to understand this, a study was done that generated a comprehensive network of 1,002 contextualized protein–protein interactions (PPIs) that are particularly related to pain. The PPIs possess an extremely coherent and interlinked structure. In this specific study, the purpose and reliability of pain-related PPIs using network have been explored *via* gene bias assessment methods. Out of the most enriched proteins in the network, majority of them play an important role in the pathology of pain for e.g., OPRM1, TPRV1, and NGF. As per the results, around 144 interactions are associated with neuropathic pain in the given dataset. Out of these 144 interactions, around 122 contribute to the pathology of pain. Neuropathic pain network contains 127 proteins out of which 8 enriched proteins are mainly involved such as GRIN2B, NOS1, MAPK14, IL-6, DLG2, CX3R1, P2RX4, and VGF. This method of utilizing disease-specific interactions presents an appreciable advancement in specificity and relevance (66).

### 1.4 Competitive Market Landscape

### 1.5 Limitations of Current Therapy

The main drawback offered by drugs used in the treatment of diabetic neuropathy was their toxicity. Hence, incorporating them into nanocarriers greatly enhanced their efficacy and reduced their toxicity. In addition, many side effects were accounted for with traditional treatment such as lack of specificity and adverse effects such as light-headedness, languidness, and multiple daily doses (68). The latest treatments do not provide adequate pain relief for about half of the patients and offer many undesired side effects such as somnolence and dizziness and the requirement of a complex dose regimen to reduce patient compliance. Standard agents for topical administration are there for the treatment of DNP, such as capsaicin cream, which is without any side effects. Still, they have low efficiency, and complex multiple administration is required, which can cause discomfort, and also the chances of contamination of sensitive body areas are also there, both of which can lead to poor patient compliance (69) (**Table 1**). The basis of this study is to incorporate novel nanotechnological approaches in mitigating DNP by targeting the DRG. Previously, opioid analgesics were widely used to treat DPN (13). Unfortunately, severe side effects were seen in patients exposed to this drug therapy. It mainly arises due to its action on the receptors present in the CNS, leading to respiratory depression, sedation, dizziness, etc. Here, nanotechnology outperforms in every aspect by delivering sensitive and targeted treatment. Another point to be taken into account is the uncontrolled drug delivery and frequent administration of drugs offered by traditional delivery systems. This probably leads to changes in plasma drug levels, thus increasing the demand for novel approaches (70).

**Table 1:** Marketed drugs for alleviating diabetic neuropathic pain (67).

S. No.	Medication	Indication	Brand name	Company	Drug class
1	Pregabalin (systemic)	DPN	Lyrica	Pfizer	Gamma-aminobutyric acid analogs
2	Topiramate (systemic)	DPN	Topamax	Mylan	Carbonic anhydrase inhibitor (anticonvulsants)
3	Duloxetine (systemic)	DPN	Cymbalta	Eli Lilly	SNRIs
4	Capsaicin cream (topical)	DPN	Zostrix, Capzasin	–	Miscellaneous topical agents
5	Carbamazepine	Neuropathic pain	Tegretol	Novartis	Dibenzapine anticonvulsants
6	Gabapentin	Neuropathic pain	Neurontin	Pfizer	Gamma-aminobutyric acid analogs
7	Nortriptyline hydrochloride or desipramine hydrochloride)	Chronic pain	Pamelor	Mallinckrodt Pharmaceuticals	Tricyclic antidepressants
8	Venlafaxine ER	DPN	Effexor	Pfizer	SNRIs

DPN, diabetic peripheral neuropathy; SNRI, serotonin–norepinephrine reuptake inhibitor.

With recent progress in identifying pain-generating processes and adopting evidence-based treatments, patients suffering from DPN are still difficult to cure. The latest treatments do not provide adequate pain relief for about half of the patients and offer many undesirable side effects such as somnolence and dizziness and the requirement of a complex dose regimen that reduces patient compliance. In addition, due to the lack of specificity of drugs, there is inadequate relief of pain. Ultimately, more understanding of the basic pathophysiological processes that lead to this complication should make it possible to devise optimal therapies for individual patients suffering from neuropathic pain (69).

### 1.6 Market Potential

Nowadays, diabetes is one of the most widespread and long-term diseases affecting most people globally (71). As per recent estimates, in the course of a year (2020–2021), the global diabetic neuropathy market is appraised to expand at a compound annual growth rate (CAGR) of 5.9%. In 2011, around 366 million people had diabetes, and the count is estimated to significantly rise to 522 million by 2030, as per approximation given by the International Diabetes Federation (72). Therefore, we can say that shortly the DNP market has stupendous opportunities to flourish. However, most of the formulations are sold by their generic names due to which there is an excellent hindrance in introducing all new and innovative therapeutic agents.

On the other hand, there has been a significant emergence and rise in the diabetic drug market after approval by the Food and Drug Administration (FDA) on using novel drugs for treating DNP. Various medications were approved, out of which two were widely used, namely, Nucynta ER and Lyrica, in 2015. The rise in the market is commonly observed in five areas, namely, Asia Pacific, South America, North America, Europe, and Africa. Among all these, North America holds the biggest market for diabetic neuropathy, where around 7.9% of adults have a chance of developing diabetes. Moreover, type 2 diabetes is directly related to obesity, hence in the US, with rising cases of obesity, there are great chances of developing DNP, thus depicting enormous market scope (73).

Hence, with comprehensive understanding about the disease, we move forward to understanding about the novel nanotechnological as well as other approaches for targeting the DRG for the treatment of neuropathic pain.

## 2 Novel Approaches for Targeting the Dorsal Root Ganglion in Mitigating Diabetic Neuropathic Pain

### 2.1 Nanoparticles

Nanoparticles represent a massive variety of particles, mainly particulate materials less than 100 nm (74). Nanoparticles exhibit remarkable and distinctive mechanized, chemical, and optical characteristics, making them a consummate agent for treating DNP. A study indicated that the CeO_2_ (cerium oxide) nanoparticles play a significant role in combating oxidative impairment and showed protective actions on diabetic neuropathy. Compared to the control group, diabetic rats showed a higher nociceptive threshold. After treatment with CeO_2_ nanoparticles, the pain threshold was reinstituted to the standard level. This study proved to be significantly successful in revealing the CeO_2_ nanoparticle as an excellent agent that suppresses nerve damage due to diabetes (75). Another study demonstrated the potential benefits of curcumin incorporated into nanoparticles in mitigating DNP arbitrated by P2Y12 receptor on SGCs in DRG. In diabetic rats, thermal hyperalgesia occurs due to modulation of IL-1 and Cx43. When curcumin nanoparticles were administered in the DRG of rats, the expression of IL-1 and Cx43 reduced significantly. Therefore, it can be said that curcumin nanoparticles are an effective therapeutic agent for treating DNP (76). One study examined the effects of emodin nanoparticles on DNP initiated by P2X3 receptors in DRG. After administration of emodin in DRG of rats, there is a significant reduction in the modulation of P2X3 receptors, thus alleviating DNP and suppressing all the channeling related to P2X3 receptors in DRG neurons (77).

#### 2.1.1 Polymeric Nanoparticles

Polymeric nanoparticles comprise nanospheres and nanocapsules, colloidal systems ranging from 10–1,000 nm in size (78). A preclinical study in rats evaluated the efficacy of baclofen-loaded PLGA nanoparticles in managing neuropathic pain. Results revealed that baclofen polymeric nanoparticles significantly reduced toxicity and increased cell feasibility on a Neuro 2a cell line. Also, in contrast to aqueous drugs, the retention time of these PLGA nanoparticles was enhanced in the brain, thus depicting it as a suitable agent in mitigating neuropathic pain (16). Bupivacaine is a local anesthetic that is commonly used to treat pain. Another study looked into the influence of bupivacaine on pain management in animals with chronic compression of the DRG. For this purpose, bupivacaine was incorporated into PLGA nanoparticles and then administered parenterally into L3 and L4 DRGs of mice. The size of nanoparticles prepared ranges from 150 ± 10 nm in diameter. Results showed that DRG administered with drug (bupivacaine) alone developed allodynia and hyperalgesia in the hind paw of mice. Whereas bupivacaine nanoparticles significantly suppressed both complications and brought the mechanical sensitivity within the range of typical values as obtained for healthy animals (17).

#### 2.1.2 Inorganic Nanoparticles

Metallic nanoparticles are composed of metals such as silver (Ag), gold (Au), and copper (Cu) along with certain metallic oxides, namely, TiO_2_ and ZnO, which impart rigid and flexible structure (79). Out of all the metals involved, silver is one of the most widely employed due to its excellent characteristics such as the large surface area-to-volume ratio (70). ROS are significant contributors to neuropathic pain. Silver nanoparticles can easily combat ROS production by binding to membrane proteins (80). Previously, many techniques were adopted to synthesize silver nanoparticles, but those methods were rejected due to toxicity of utilized chemicals. This led to the idea of employing medicinal plants in the development of silver nanoparticles (81). One study involved *Nigella sativa* extracted in the green synthesis of silver nanoparticles and determined its beneficial effects in diabetic neuropathy. An experiment was carried out in which a healthy control group of rats was compared to the diabetic neuropathy-induced group to estimate the potential actions of nanoparticles administered. Results revealed that neuropathy-induced group showed significant demodulation in brain tropomyosin receptor kinase A (trKA) levels and increased inflammatory mediators. However, the group treated with silver nanoparticles experienced less pain and enhanced retention time. Thus, due to its antidiabetic, anti-inflammatory, and antioxidant effects, silver nanoparticles combined with *N. sativa* could be an innovative treatment option against diabetic neuropathy (82).

#### 2.1.3 Gene-Based Nanoparticles (siRNA)

siRNA is a double-stranded RNA molecule that causes obtrusion in the genetic expression of complementary base pairs of mRNA and leads to knockdown of expression (83). Microglia homing peptide molecules are sound delivery systems for siRNA due to their potent knockdown efficacy. One of the most frequently employed homing peptides for the siRNA–interferon regulatory factor-1 complex is MG1. Compared to standard siRNA and other peptide molecules, the siRNA delivery system was eminent in reducing hyperalgesia-associated nerve damage. Such shreds of evidence suggest siRNA delivery candidates as a plausible therapeutic in alleviating neuropathic pain. Calcitonin gene-related peptide located in the DRG primarily impacts nociception in afferent transmission input. This activation of afferent neurons leads to the release of the calcitonin gene-related peptide in the spinal cord. Due to glutamate release, NMDA receptors activate, which further enhances calcium influx in the cell, triggering the release of more calcium from stores. As a result, enhanced calcium ion levels actuate various protein kinases involved in the pathophysiology of neuropathic pain (55). siRNA delivery device mitigates neuropathic pain by suppressing the P2X_3_ receptor in the DRG and leads to inhibition of expressed Calcitonin gene-related peptide in the spinal cord (84). There is the release of numerous cytokines, which stimulates kinase activated process. siRNA delivery device halts this activation thereby, alleviating neuropathic pain (61).

**Figure 3:**
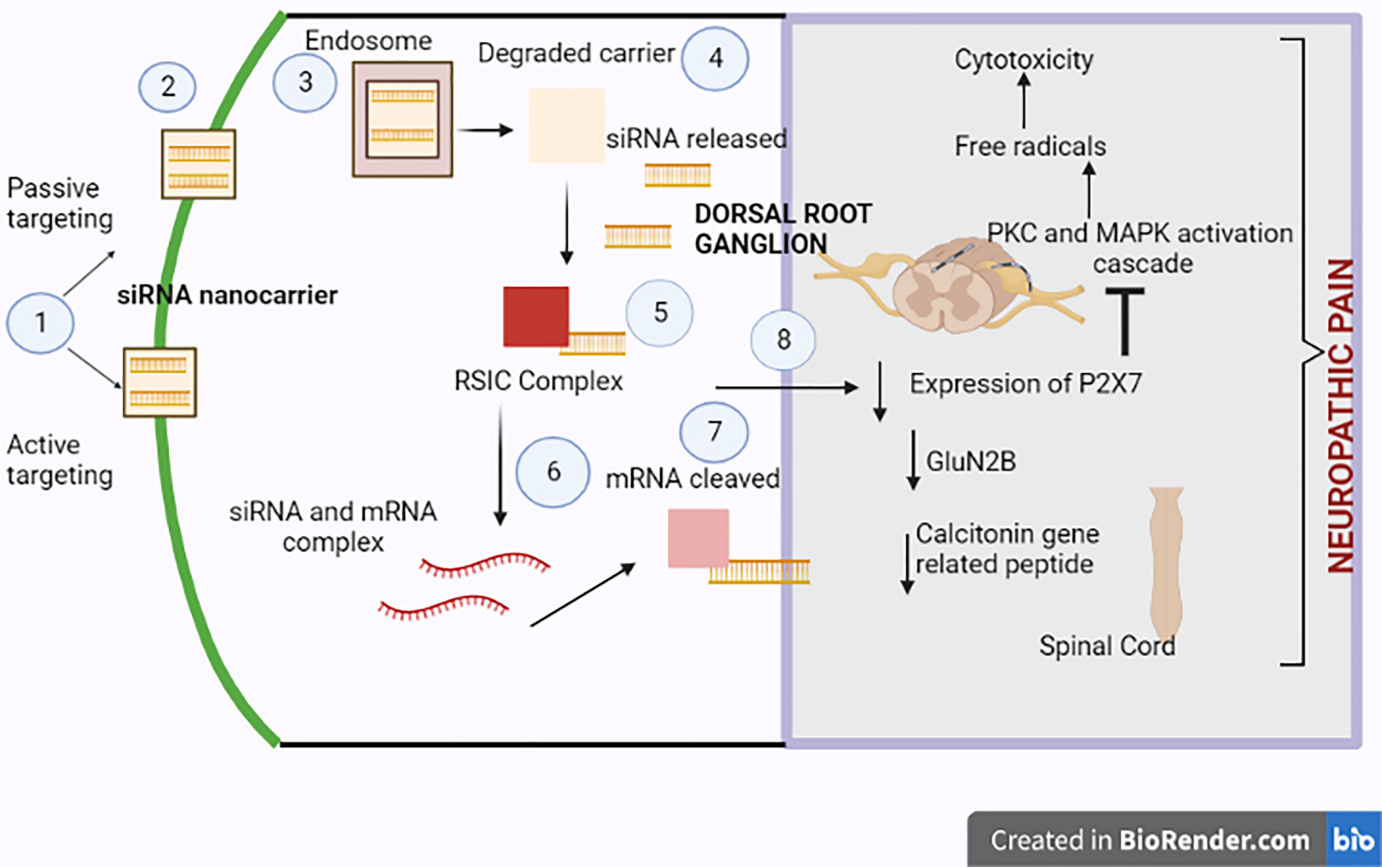
Neuropathic pain-relieving molecular mechanism of siRNA-based nanocarriers. 1) Either passive or active targeting allows the siRNA delivery device to penetrate the cell. The attachment of antibodies or aptamers, which improve the device’s specificity, aids in active targeting. 2) The siRNA nanocarrier then enters the cell. 3) There is the engulfment of the delivery device by the endosome. 4) As a result, the outer carrier breaks down, releasing free siRNA therapeutics. 5) An RNA-induced silencing complex (RISC) is formed due to the siRNA formation. 6) To progress the knockdown of the chosen mRNA, the mRNA and siRNA interact with one another. 7) The RISC cleaves the mRNA to silence proteins implicated in neuropathic pain disease. 8) P2X7 receptor expression in the dorsal root ganglion, GluN2B peptide, and the calcitonin gene-related peptide in the spinal cord are suppressed. The siRNA delivery device reduces neuropathic pain by inhibiting excitation transmission through the P2X3 receptor in the DRG and inhibiting expressed calcitonin gene-related peptide in the spinal cord, which changes the calcium-augmented pathways in neuropathic pain.

Lentivirus-containing siRNA was introduced into the spinal cord *via* the intrathecal route in a rat model. The results revealed a diminution in nociception due to the sequential inhibition of mRNA and expressed protein GluN2B. Furthermore, the lentiviral delivery device successfully introduced GluN2B to the dorsal horn, thus reducing neuropathic pain (85). **Figure 3** corresponds to the mechanism of siRNA-based nanocarriers in alleviating neuropathic pain.

#### 2.1.4 Solid Lipid Nanoparticles and Nanostructured Lipid Carriers

Solid lipid nanoparticles (SLNs) promise drug delivery systems that consist of solid lipid particles such as fatty acids and waxes to which surface-active agents have been added to form a stable matrix system. SLNs can easily inculcate both hydrophilic and hydrophobic drugs in their matrix, thus preventing the medicine from any deterioration (79). However, SLNs possessed some drawbacks due to which concept of nanostructured lipid carriers (NLCs) came into existence. NLCs outperform SLNs in terms of good drug loading capability (78). Capsaicin is widely used in treating DNP due to its ability to bind to TRPV1 present on Aδ and C-nerve fibers (86). Capsaicin (0.25%)-loaded lipid nanoparticles were developed from capsicum extract in the study. There was strategic incorporation of capsaicin into SLNs and NLCs without any toxic solvent involvement. The particle size of prepared nanoparticles was less than 200 nm. Compared to SLNs, NLCs offer enhanced encapsulation capability and better capsaicin liberation, thereby augmenting its release into deeper skin layers. Hence, through the above study, capsaicin-loaded lipid nanoparticles could be an excellent therapeutic agent for pain management (87).

### 2.2 Nanoemulsion

One of the highly recommended drug delivery systems, nanoemulsion, consists of oil, surfactant, and water in relevant ratios. Nanoemulsion bears an average atom size of 1–100 nm (68). These are widely used (88). Various experiments have been conducted to determine the potential of nanoemulsion incorporating *Bauhinia variegata* to treat diabetic peripheral neuropathic pain *via* the acupuncture technique. Due to its polyphenol and flavonoid content, *Bauhinia* exhibits free radical-scavenging properties. Experimental rats were administered streptozocin, employing intraperitoneal injection to induce diabetes. Administration of *Bauhinia variegata* nanoemulsion normalized blood glucose levels compared to the control group. Long-term treatment with nanoemulsion effectively reduced hind paw abolition latency and alleviated allodynia. Therefore, through the above experiment, one can presume that *Bauhinia* nanoemulsion could effectively relieve peripheral neuropathic pain (89). Furthermore, α-eloestearic acid, one of the main constituents of the bitter gourd when administered orally in the form of nanoemulsion to diabetic rats, showed promising effects by reducing neuropathic pain (90).

### 2.3 Liposomes

Liposomes are the most widely used nano delivery system, as they can significantly increase drug efficacy while minimizing their side effects. Liposomes consist of an aqueous core encircled by phospholipid bilayers (91). Ropivacaine is a widely used anesthetic for mitigating neuropathic pain (92). To alleviate long-term neuropathic pain, it was seen that liposomal preparation of ropivacaine (Rop-DPRL) could lead to the cytotoxicity of cancerous cells *via* nutrient destitution. Another study demonstrated the effects of zoledronic acid (ZOL) in mitigating neuropathic pain. The most pronounced drawback of ZOL is its pharmacokinetic outline. Hence, an animal model developed and assessed ZOL incorporating PEGylated liposomes (LipoZOL) for its action in attenuating neuropathic pain. There is partial or complete disorganization of the blood–brain barrier (BBB) in chronic neuropathic pain, which permits the safe entry of nanocarriers such as LipoZOL. Changes in BBB due to sciatic nerve destruction encourage the invasion of LipoZOL in the dorsal horn of the spinal cord, thereby administering adequate concentrations of ZOL in the CNS. This further regulates the phenotypical shift of glial cells, thus alleviating neuropathic pain (93).

### 2.4 Exosomes/Extracellular Vesicles

Exosomes are small vesicles that are seen in body fluids. Exosomes are acknowledged for their excellent capacity for loading nucleic acids and are less toxic than other novel carriers such as carbon nanotubes and fullerenes (94). They are primarily apprehended for their enhanced action, as they serve as carriers for numerous molecules, including proteins, nucleic acids, and lipids. As we know, RNAse leads to the destruction of miRNA, so it was loaded into extracellular vesicles to prevent its degradation. These exosome-loaded miRNAs impact physiological responses in beneficiary cells by controlling gene expression (95). Superoxide dismutase (SOD)-loaded polymersomes are highly beneficial in treating neuropathic pain, as they possess antioxidant action. These SOD-loaded polymersomes have several advantages such as appropriate interaction of the enzyme with ROS due to porous membrane and enzymes maintained their original shape. Treatment with SOD-loaded polymersomes is much effective for treating neuropathic pain as compared to SOD alone after painful DRG compression (96).

## 3 Ligand-Based Targeting to Dorsal Root Ganglion

Recently, the concept of small peptide aptamer is gaining a lot of undivided attention in treating neuropathic pain as these target protein–protein connections in pain pathways. Also, these aptamers have been considered as a useful clinical tool in alleviating chronic pain (97). Compared to gene delivery strategies such as RNA interference, this peptide aptamer hindrance can effectively slab various interactions selectively, causing functional knockdown. Ca^2+^ channel-binding domain 3 (CBD3), in association with the TAT motif (TAT-CBD3), is a famous example of a peptide inhibitor that can prevent the pain caused by different conditions. However, after proving its excellent efficacy results, CBD3 in conjunction with TAT has been utilized widely as an alternative to mitigate chronic pain (98). A study suggested that Adeno-associated viruses (AAV) injection can lead to uninterrupted CBD3 expression in DRG neurons, relieving pain with reduced or no toxic effects. Here, voltage-gated Ca^2+^ channels (VGCCs) were selected as molecular targets, as they play a significant role in synchronizing neuron excitability and transmission *via* synapses (99). Ultimately, small interfering peptides can be utilized as an effective alternative and strategy for treating neuropathic pain (97). One study suggested that chemokine receptor CXCR3 is involved in generating chronic pain. It is present in spinal cord, and the pain is generated due to mast cell destruction due to which there is release of histamine. In this situation, histamine antagonists H1 and H4 can be used as plausible ligands to stop the release by blocking CXCR3 receptors (100).

## 4 Other Approaches

### 4.1 Neuromodulation

Neuromodulation is a rapidly emerging area of pain medicine that influences hundreds of thousands of patients dealing with several disorders globally (101). It involves the utilization of noninvasive and surgical electrical therapies. In the case of PDN, neuromodulation seems to be a very effective treatment option for those patients who are generally insensitive to conventional pharmacotherapy (102). Therefore, the exemplary treatment method, namely, tonic spinal cord stimulation (t-SCS), has been incorporated. It mainly involves the entry of regular electrical pulses into the dorsal column *via* epidural electrodes. The electrical pulses are delivered at a frequency of around 50 Hz (103). DRG stimulation or neuromodulation can effectively cause a reduction in chronic pain associated with PDN (104). DRG and DNP-DRG may be particularly susceptible to this disease for several reasons such as the following:

a) DRG consists of sensory neurons, which are not protected from the BBB, and the ambient oxygen tensions in DRG are pretty low. These physiological conditions may suggest that these may be vulnerable to microangiopathy, which is a complication related to diabetes (105).

b) Also, the involvement of sensory neurons in early diabetic polyneuropathy may put forward the fact that diabetes specifically targets DRG. Certain features associated with DRG might imply that it would be exposed to changes known to occur in diabetes such as excessive polyol flux, microangiopathy, and protein glycosylation (106).

c) Several studies, including streptozocin-induced DPN, also highlighted the fact that DRG is closely related to painful DPN acquiring several metabolic and immunological processes (107).

d) Certain receptors such as TRPV1 present in DRG are closely associated with DNP (108).

### 4.2 Precision Medicine

Several techniques are available to mitigate DNP, but neither glucose control nor the symptomatic treatment is very successful in doing so. Therefore, to overcome this issue, a concept has been taken into account that hypothesizes the study of patient characteristics. The concept could possibly be helpful to stratify individuals, thus providing them specific and targeted therapy to get better pain relief. This whole concept of studying patient characteristics [clinical features, quantification sensory testing (QST), genetics, and cerebral imaging] and then developing targeted therapy is termed “precision medicine” (109).

## 5 Conclusion

Diabetic neuropathy is the most entrenched complication of diabetes. Typically, it affects the distal foot and toes, gradually approaching the lower part of the legs. Diabetic foot ulcer (DFU) could be one of the worst complications of DM. Long-term diabetes leads to hyperglycemia, which is considered to be the utmost contributor to neuropathic pain. Therefore, using antidepressants, GABA analogs, opioids, and topical agents to treat pain in PDN is recommended. Currently available systemic medications provide adequate pain relief for approximately half of affected patients and are limited by unwanted adverse reactions and multiple-dose regimens. So, other treatment options need to be explored to treat this widespread complication of diabetes, mainly involving novel nanotechnological approaches. Nanotechnology plays a significant role in effectively delivering drugs (analgesics) to a specific site, thus mitigating chronic pain. Some of the standard painkillers, namely, baclofen, bupivacaine, and morphine, were formulated with liposomes, polyesters, PLGA, and nanoemulsions, etc., to improve their efficacy. siRNA can also be used as potential therapeutics to treat DPN but are limited by its unstable nature under normal physiology in the blood, wherein it undergoes digestion by nuclease enzymes. Therefore, innovative nanotechnological approaches such as liposomes, niosomes, nanoemulsions, SLNs, and NLCs have been utilized to overcome conventional therapy’s drawbacks.

## 6 Future Perspectives

Despite having so many alternative therapeutic options for treating DNP, still, pharmacological treatment remains a big never-ending issue for physicians. Therefore, there is a need to find out various important target areas that can be utilized directly to mitigate DPN. We can also expect multiple novel nanotechnology-based products in the market to treat diabetic neuropathy, which can adequately manage the condition with enhanced effects. Moreover, natural plant-based products are also being studied to a large extent to provide more safe and cheap treatment to the patients. Furthermore, many advancements have been made about gene therapy, including new therapeutic approaches that may become combination therapies with various siRNAs targeting various survival pathways or a combination of specific siRNAs that may sensitize the treatment of DNP with other pain-relieving drugs. Therefore, in association with novel technological approaches, conventional medicines can significantly enhance their action toward diabetic neuropathy, and we can expect plenty of nano-based products in the market for the mitigation of diabetic neuropathy.

## Author Contributions

RB: conceptualization, methodology, writing—review, editing and visualization, literature search. AS: literature search, data collection, and writing. AK: final supervision. All authors contributed to the article and approved the submitted version.

## Funding

The authors acknowledge the funding received by AK & RB from DST-UT Chandigarh grant-2020 (No. S&T&RE/RP/147/e-2873/Sanc/02/2021/1154-1161 for the project entitled “A novel healthcare solution for diabetes and cancer neuropathic pain patients”.

## Conflict of Interest

The authors declare that the research was conducted in the absence of any commercial or financial relationships that could be construed as a potential conflict of interest.

## Publisher’s Note

All claims expressed in this article are solely those of the authors and do not necessarily represent those of their affiliated organizations, or those of the publisher, the editors and the reviewers. Any product that may be evaluated in this article, or claim that may be made by its manufacturer, is not guaranteed or endorsed by the publisher.
